# Medical Society of the County of Chautauqua

**Published:** 1896-08

**Authors:** Charles A. Ellis

**Affiliations:** Secretary


### Society Proceedings.

### MEDICAL SOCIETY OF THE COUNTY OF CHAUTAUQUA.

Reported by CHARLES A. ELLIS, M. D., Secretary.

THE annual meeting of the Medical Society of the County of Chautauqua was held at the Hotel Atheneum, Chautauqua, N. Y., Tuesday, July 14, 1896.

The president, Dr. E. S. Rich, of Kennedy, called the society to order at 11 o'clock A. M. for the transaction of business.

The following-named were elected officers for the ensuing year: President, E. S. Rich, M. D., Kennedy; vice-president, M. N. Bemus, M. D., Jamestown; secretary and treasurer, C. A. Ellis, M. D., Sherman; delegates to the Medical Society of the State of New York, C. A. Ellis, M. D., and E. S. Rich, M. D.

AFTERNOON SESSION, 2 O'CLOCK.

President's address. Management of chronic valvular diseases of heart, Dr. Chas. G. Stockton, Buffalo, N. Y. A study of some phases of tuberculosis, Dr. A. D. Lake, Gowanda, N. Y. General discussion. Anesthesia in obstetrics, Dr. J. W. Morris, Jamestown, N. Y. Discussion, Dr. J. H. Wiggins, Jamestown, N. Y. Erysipelas, Dr. M. N. Bemus, Jamestown, N. Y. Criminal abortion, Dr. James Murphy, Sherman, N. Y. Discussed by Dr. T. D. Strong, Westfield, N. Y.; Dr. E. A. Scofield, Bemus Point, N. Y.

Dr. V. M. Griswold, of Fredonia, called attention to the fact that hydrochlorate of quinine was a safe and efficient local anesthetic when used hypodermatically, but he stated that it was not absorbed by mucous surfaces. He had been using it for some time and considered it much safer than cocaine.

The following named physicians were present as guests of the society: Drs. Chas. G. Stockton, Lucien Howe and C. C. Frederick, of Buffalo; Dr. Gould, of Philadelphia; Dr. Seaver, of Yale College, and Dr. Bainbridge, of the Presbyterian Hospital, New York City.

Upon invitation of the management of the Chautauqua Assembly, a number of ladies, wives and relatives of the members, were present, and through the courtesy of the assembly the visiting cards of physicians were received as admission tickets to the grounds for two days, accompanying ladies included.

In the evening a banquet was given at the Hotel Atheneum, at 8.30 o'clock, at which the ladies were present. Prof. Geo. E. Vincent, of Chicago, acted as toastmaster, when the following list of toasts was offered:

The Chautauqua County Medical Society—its past and present, Dr. T. D. Strong, Westfield. The press, Mr. Fred. W. Hyde, Jamestown. The pedagogue, Supt. W. L. MacGowan, Warren, Pa. The physician's wife, Mrs. O. C. Shaw, Kennedy. Chautauqua, Prof. Geo. E. Vincent, Chicago. Vocal solo, Mrs. Morris N. Bemus, Jamestown.

This was one of the most interesting and successful meetings ever held by this society and should tend to stimulate renewed interest in its proceedings in the future.

